# The Use of Low-Calorie Sweeteners by Children: Implications for Weight Management^1^,^2^,^3^

**DOI:** 10.3945/jn.111.149609

**Published:** 2012-05-09

**Authors:** John Foreyt, Ronald Kleinman, Rebecca J. Brown, Rachel Lindstrom

**Affiliations:** 4 Baylor College of Medicine, Houston, TX; 5 Harvard Medical School, Massachusetts General Hospital, Massachusetts General Hospital for Children, Boston, MA; 6 National Institutes of Health, Bethesda, MD; 7 America on the Move Foundation, Aurora, CO

## Abstract

The rise in pediatric obesity since the 1970s has been well established in the United States and is becoming a major concern worldwide. As a potential means to help slow the obesity epidemic, low-calorie sweeteners (LCS) have gained attention as dietary tools to assist in adherence to weight loss plans or prevention of excess weight gain. Observational studies tend to show positive correlations between LCS consumption and weight gain in children and adolescents. Although the data are intriguing, these epidemiologic studies do not establish that LCS cause weight gain, because there are likely many lifestyle and genetic differences between children and families who choose to consume LCS and those who do not. Short-term randomized controlled trials have shown LCS use to be BMI neutral or to have modest weight-reducing effects in overweight and obese adolescents. The long-term effects of LCS in children and adolescents are unknown. Some compelling research is currently underway and may provide needed insight into the potential role of LCS in weight management. The paucity of data regarding the effects of LCS use in children and adolescents creates challenges in decision-making for health care providers and parents.

## Introduction

Obesity has become a highly prevalent issue in virtually every area of the world. Although genes play a role in governing energy expenditure as well as energy intake, most agree that the current environment in both resource-poor as well as developed countries has supported an increasing prevalence of overweight and obesity in children and adults (1, 2). We have come to understand gestation, infancy, and early childhood are zones of opportunity for intervention during childhood. The prevalence of overweight and obesity among children in the United States has been increasing for the past 50 y. However, from infancy to age 5 y, the prevalence seems to be stable when measured over the past 10 y (1). This is not the case for older children and adolescents, in whom the prevalence of a BMI greater than the 85th percentile continues to increase and is now >30%. This has important implications for the life expectancy of children today and raises the prospect of a population of children who may have a shorter life expectancy than their parents (3).

This expectation of a shortened lifespan is a result of the comorbidities associated with overweight and obesity that can affect virtually every organ system in the body, similar to those seen in adults. Type 2 diabetes is one complication of obesity in childhood. More than 15% of new diabetes cases in children are now type 2 diabetes and this is largely related to the increase in obesity (4). Another complication of diabetes is fatty liver disease. The incidence of fatty liver disease, including steatosis and steatohepatitis, is 15–20 times higher in obese adolescents compared with lean adolescents (5). Dyslipidemia, yet another complication, is also commonly found among obese children. In fact, >70% of obese children (ages 2–17 y) have at least one additional cardiovascular risk and 30% have ≥2 cardiovascular risks (6). Forty-three percent of obese adolescents aged 12–19 y are candidates for lipid screening and lifestyle counseling (7).

There are also sizeable implications for medical costs as a result of obesity in childhood. Annual pediatric hospital costs rose from $35 million during 1979–1981 to $127 million during 1997–1999, a 4-fold increase. Pediatric admissions to the hospital in the 20-y period from 1980 to 2000 rose 15% for diabetes, 55% for obesity as a whole, 175% for sleep apnea, and 7% for gallbladder disease (8).

A recent survey showed that physicians remain highly trusted sources of child health information for parents (9). Clinicians strongly agree that it is important to intervene to counter childhood obesity among their patients (10). To that end, the American Academy of Pediatrics (AAP)^8^, along with many other national and international organizations, has developed action plans to prevent and treat obesity in the health care setting. Current recommendations by the AAP for point-of-care guidance include measuring BMI and/or weight-for-length at every visit to the health care provider, providing age-appropriate nutritional and physical activity guidance at every visit, and delivering the following messages: for diet, 5 fruits and vegetables per day; for activity, 1 h of physical activity every day; for screen time/inactivity, no more than 2 h each day; no sugar-sweetened drinks; and support of breastfeeding. Growth charts to plot BMI, weight, and length are available from the CDC and WHO. Current recommendations from the CDC and the AAP are to use WHO growth charts from birth through 23 mo of age and thereafter to use the CDC reference standard growth charts (11, 12). However, understanding how to interpret these charts is not universal among parents. In a recent survey, 80% of parents had seen a growth chart and most thought that they understood it well; the majority also thought it was important to be shown growth charts to see how their child was growing. However, only 64% of parents could identify a child’s weight when shown a plotted point on a growth chart, only 56% could identify the definition of percentile, and almost 80% incorrectly interpreted charts containing height and weight measurements in tandem (13). Therefore, it is clear that although pediatricians almost universally use these charts, there is a gap in parental understanding of the implications of measurements plotted on these charts. In another survey, 66% of parents whose child’s weight was in the top quartile preferred that their child weighed that much and when viewing hypothetical infant growth trajectories, ~30% chose charts showing infants at the 90% percentile for weight at age 1 y as being the healthiest (14). Even more important is a need to recognize the increased risk associated with upward crossing of major weight-for-length percentiles in the first 6 mo of life. Contrary to widely held beliefs, upward crossing of 2 major weight-for-length percentiles is associated with a high risk of obesity 5 and 10 y later (15). Thus, an effort to curb excess weight in infancy may be particularly useful in preventing childhood obesity and its consequences. This makes an understanding of plotted measurements on growth charts even more important.

There are a number of barriers that health care providers encounter in obesity prevention and treatment. These include the lack of insurance reimbursements, lack of time during routine office visits, lack of knowledge about obesity and recommendations for an approach in the office setting, lack of resources in the community for treatment, and parent disinterest or misperceptions. Among low-income mothers in Mexico, 43% underestimated their child’s weight status and this rose to >80% of mothers of overweight or obese children (16). A high percentage of the mothers at these clinics wish that their children were less active and most did not consider that sugar-sweetened beverages (SSB) and high-fat snacks might be inappropriate for their children’s health (16). In a recent article published in the *Journal of the American Dietetic Association*, a cross-sectional analysis of almost 2400 children aged 1–5 y living in Greece found that a higher parental BMI and rapid infancy weight gain were the main determinants of obesity in preschool years and, as in other surveys, maternal underestimation of children’s weight status was more likely for children with rapid gain in infancy (17). In a review of the literature on parent perceptions of their child’s overweight, parents of overweight children consistently underestimated their child’s weight status or were not concerned about the risks associated with an overweight child (18). Age, gender, and ethnicity often influence parent perception of child overweight and parents use criteria other than growth charts to perceive overweight as a problem. Another recent survey showed that although mothers generally recalled that growth charts were used and were able to recall their child’s height and weight percentiles, they were usually unable to articulate the meaning of these percentiles (18). More importantly, most mothers stated that their nutrition-related decisions were not influenced by growth chart findings. Interestingly, a considerable proportion of mothers reported that nutrition was not discussed at the most recent well-child visit (18).

All of these findings reinforce the importance of counseling during the course of a visit and a discussion of weight, growth, nutrition, and physical activity. The AAP introduced a program known as Let’s Move and has put its recommendations in the form of a prescription for healthy active living. This program includes a number of electronic resources available for use in the office or clinic setting (19). Thus, this approach is intended to prevent overweight in childhood and emphasizes behavioral modification that involves the whole family by implementing the diet and activity recommendations in Let’s Move (each day: 5 fruits and vegetables; no more than 2 h of screen time; 1 h of moderate to vigorous physical activity; 0 sweetened beverages). With this approach and the available resources to support them, as well as a commitment by national organizations to focus on infancy, childhood, and adolescence as opportune times to intervene and prevent the development of obesity by encouraging a healthy diet and an active lifestyle, we may see that the current trends among children aged 0–5 y persist and even extend into adolescent years and adulthood.

## Low-Calorie Sweetener Use for Weight Management in Children: Benefits and Considerations

Low-calorie sweeteners (LCS) approved for use by the FDA include acesulfame potassium, aspartame, neotame, saccharin, stevia, and sucralose (20). Although widely varying in chemical structure, these compounds have in common the ability to stimulate the human sweet-taste receptor (21) while providing zero to minimal calories. As a potential means to help curtail the obesity epidemic, LCS have gained attention as dietary tools (22) that provide sweet taste without the extra energy derived from foods and beverages containing energy-containing sweeteners (23–27) and thus may assist in adherence to weight loss plans or prevention of excess weight gain (28). In contrast, epidemiologic studies showing an association of LCS use with increased body weight have raised concern that LCS use may have adverse consequences in obesity prevention and treatment (29). The paucity of data regarding the effects of LCS use in children creates challenges in decision making for both health care providers and parents. The unique developmental changes of childhood, during which taste preferences and eating habits develop, both raise the stakes and increase the difficulty of research in this field. Although fewer children consume LCS compared with adults, ~15% of the U.S. population >2 y of age uses LCS (30), and the proportion of children consuming LCS is growing (20). In this section, we review the existing evidence for effects of LCS on food intake and body weight in children and discuss important unanswered questions about appropriate LCS use in children.

### Observational studies of LCS and weight gain in children.

Nine observational studies (including >20,000 children) have examined the relationship between LCS consumption (typically measured as consumption of beverages containing LCS) and outcomes such as weight gain or obesity (**Table 1**). Similar to data in adults, the majority of pediatric epidemiologic studies have shown a positive correlation between weight-related outcomes and LCS beverage (LCSB) intake, meaning that children who consumed more LCS were more likely to eat more, gain excess weight, or be obese. Two of 3 cross-sectional studies (31, 32) showed positive associations between LCSB intake and BMI, whereas one study showed no such association in younger children (aged 2–5 y) (33). Of the 6 longitudinal studies, 4 showed positive associations between LCSB intake and changes in weight (in boys, but not girls) (34), BMI Z-score (35), energy intake (36), and fat mass, although the latter was no longer significant after adjustment for covariates (37). A single study showed that increased consumption of LCSB was correlated with lower odds of obesity (38) and one study showed no association between change in LCSB intake from age 3 to 6 y and change in BMI Z-score (39). Although these data supporting an association between LCS consumption and weight gain are intriguing, epidemiologic studies cannot establish that LCS cause weight gain, because there are likely many lifestyle and genetic differences between children and families who choose to consume LCS and those who do not. For example, obese parents may choose to offer their child foods or drinks containing LCS because they believe that their child is at increased risk for developing obesity. Thus, a cautious conclusion that can be drawn from such studies is that LCS use, as practiced by the participants in these studies (with their various genetic backgrounds and lifestyle choices), was not an effective strategy in preventing excess weight gain.

**TABLE 1:** Observational studies of LCS use in children^1^

Reference	*n*	Participants (age; sample; year)^2^	Duration	Results
Cross-sectional studies				
Forshee et al. (31)	3311	6–19 y; population-based (US); 1994–1996, 1998	—	BMI was positively associated with LCSB consumption after adjustment for age, race, and family income
Giammattei et al. (32)	385	11–13 y; school-based (Santa Barbara, CA); 2000–2001	—	BMI Z-score and percentage fat were positively associated with LCSB consumption (without adjustment for sociodemographic variables)
O'Connor et al. (33)	1160	2–5 y; population-based (US); 1999–2002	—	No association was found between LCSB consumption and BMI
Prospective cohort studies				
Ludwig et al. (38)	548	11.7 ± 0.8 y; school-based (Boston, MA); 1995, 1997	19 mo	BMI was not associated with either baseline or change in LCSB intake, but incident obesity was negatively associated with change in LCSB intake (adjusted for baseline BMI, triceps skinfold thickness, age, gender, ethnicity, other dietary variables, physical activity, television viewing, and total energy intake)
Berkey et al. (34)	11654	9–14 y; Children of Nurses’ Health Study II (US); 1996, 1997, 1998	2 y	LCSB intake positively associated with BMI change in boys, but not in girls [adjusted for age, Tanner stage, race, menarche, prior BMI Z-score, linear growth, milk type (whole/2%/1%/nonfat/soy), physical activity, and inactivity]
Blum et al. (35)	166	9.3 ± 1 y; school-based (Nebraska); 1992–1996	2 y	LCSB intake at y 2 was positively associated with BMI Z-score at y 2 (adjusted for baseline BMI Z-score)
Striegel-Moore et al. (36)	2371	9–10 y; convenience/random sample of black and white girls (3 U.S. sites); 1987–1997	10 y	LCSB intake was positively associated with total daily energy intake, but not BMI (adjusted for study site, race, and other beverage consumption)
Johnson et al. (37)	1203	5 y; population-based (UK); 1997–2002	4 y	LCSB consumption at ages 5 and 7 y positively associated with fat mass at age 9 y (this was no longer significant after adjustment for baseline BMI, television viewing, maternal education, paternal class, parental BMI, misreporting of energy intake, dietary energy density, percentage dietary fat, and fiber density)
Kral et al. (39)	177	3 y	3 y	No association between change in LCSB consumption and change in BMI Z-score

1 LCS, low-calorie sweetener; LCSB, low-calorie sweetened beverage.

2 Ages are at study entry.

### Acute effects of LCS on food intake in children.

Seven small studies have examined the acute effects of LCS on food intake (**Table 2**). These studies included between 14 and 262 children (374 total) aged 3–14 y. All of these studies use a similar design. The child first consumes a “preload,” which is a food or drink sweetened with an energy-containing sugar, a LCS, or no sweetener at all (e.g., water). After the child consumes the preload, there is a time delay (0–90 min in these studies), after which the child is offered an ad libitum meal and the amount of energy consumed is measured. This type of study has the potential to answer questions about how the use of LCS in snacks or beverages affects overall energy intake over a period of hours. However, small variations in study design substantially influenced the results. For example, when the preload was consumed 0–30 min prior to the ad libitum meal, LCS (vs. energy-containing sweeteners) had no effect on total energy intake (40, 41). In contrast, when the preload was consumed 60–90 min prior to the ad libitum meal, LCS (vs. energy-containing sweeteners) reduced total energy intake (42–45). Only one small study (44) compared LCS with unsweetened water; the results were even more complex, with LCS reducing total energy intake when consumed 30 min before a meal, but not 0 or 60 min prior. The largest study, including 262 children aged 5–12 y, showed that LCS had little effect on total energy intake in the youngest children (ages 5–6 y) but that LCS reduced energy intake in older children (ages 9–12 y) (46). Taken together, these data suggest that if LCS were used to replace sugar-sweetened foods and drinks, they might reduce energy intake over a period of hours in older children if consumed between meals, but might not affect energy intake if consumed with meals. It is important to note, however, that the behavior of children in a laboratory setting may not reflect their behavior in real life and that any reduction in energy intake over a period of hours attributable to LCS may be compensated for with increased energy intake in subsequent hours or days. Thus, these studies provide little insight into the effects of substituting LCS for energy-containing sweeteners in children’s diets on long-term energy intake and weight gain.

**TABLE 2:** Controlled trials of the acute effects of LCS on food intake^1^

Reference	*n*	Participants^2^	Design	Intervention	Method of randomization	Results
Birch et al. (45)	18	3–5 y	Crossover	2 sessions each of aspartame-sweetened (low-energy) vs. maltodextrin-sweetened (high-energy) pudding, followed by ad libitum snack; final session with intermediate-calorie pudding followed by ad libitum snack	Not specified	Snack consumption after intermediate-energy pudding was greater (by ~50 kcal) when its flavor was that previously paired with low-energy pudding vs. flavor previously paired with high-energy pudding
Birch et al. (42)	22 children, 26 adults	2.5–5 y, 25–35 y	Crossover	Aspartame- vs. maltodextrin-sweetened pudding, followed by ad libitum snack	Not specified	Children had ~100% compensation^3^ in ad libitum snack 20 min after aspartame (low-energy) vs. maltodextrin (high-energy) sweetened pudding; adults showed no compensation (~0%)
Birch et al. (43)	24	2–5 y	Crossover	Aspartame-sweetened, sucrose-sweetened, or unsweetened drink, followed by ad libitum snack	Not specified	60% compensation in ad libitum food intake 0 min after aspartame- vs. sucrose-sweetened preload, but ~0% compensation 30 or 60 min after preload; children given aspartame-sweetened preload vs. water reduced ad libitum intake at 30 min but not at 0 or 60 min
Anderson et al. (44)	20	9–10 y	Crossover	Aspartame- or sucrose-sweetened drink, followed by ad libitum lunch	Not specified	6% compensation in ad libitum lunch intake 90 min after aspartame- vs. sucrose-sweetened preload
Johnson et al. (46)	262	5–12 y	Crossover	Aspartame- or sugar-sweetened drink, followed by ad libitum lunch	Not specified	49% compensation overall; compensation decreased with age and did not vary by ethnicity (non-Hispanic white vs. Hispanic) or gender
Bellissimo et al. (40)	14	9–14 y boys	Crossover	Sucralose- or glucose-sweetened drink, followed by ad libitum pizza lunch	Not specified	94% compensation in ad libitum lunch intake 30 min after sucralose- vs. glucose-sweetened preload
Bellissimo et al. (41)	14	9–14 y boys	Crossover	Sucralose- or glucose-sweetened drink, followed by ad libitum pizza lunch	Not specified	112% compensation in ad libitum lunch intake 30 min after sucralose- vs. glucose-sweetened preload; 66% compensation if watching TV during lunch

1 LCS, low-calorie sweetener.

2 Ages are at study entry.

3 Compensation after a preload is defined as the difference in subsequent ad libitum energy intake between 2 conditions, divided by the energy in the preload.

### Short-term effects of LCS on weight in children.

Interventional studies, in which children are randomized to receive or not receive LCS, have the potential to provide better evidence for a causal relationship between LCS and health outcomes. Four small studies (including 374 children) looked at the effects of LCS on body weight during 12–25 wk (**Table 3**). The earliest study showed no effect of 2.7 g/d of encapsulated aspartame (vs. placebo) on weight loss in 55 overweight, 10- to 21-y olds receiving a 13-wk, 1000-kcal/d diet (47). It is worth noting that 2.7 g of aspartame is equivalent to that found in fifteen 12-oz (355 mL) diet sodas and approaches the acceptable daily intake for a 70-kg adult. A 2006 study examined the effect of reducing SSB by replacing them with LCSB or water for 25 wk (48). The intervention had no effect on BMI for the entire cohort of 103 participants aged 13–18 y, but a post hoc subgroup analysis demonstrated benefit in the heaviest participants. A larger study testing this intervention in overweight adolescents is ongoing.^9^ A similar study published in 2007 showed that permitting SSB compared with permitting only LCS-sweetened drinks did not affect weight loss in 32 adolescent girls (23). The America On the Move study (discussed below) showed modest beneficial effects of a 2-pronged approach of both increased activity and reduction of energy intake using the LCS, sucralose, in 184 overweight children aged 7–14 y (49). However, due to the dual nature of the intervention, the effect of sucralose cannot be isolated in this study. Overall, these short-term, randomized controlled trials (RCT) suggest that LCS are BMI neutral or minimally reduce BMI in overweight and obese adolescents; however, more data are needed prior to making blanket recommendations regarding use of LCS for weight in children and adolescents.

**TABLE 3:** Short-term interventional studies on LCS use in children: RCT^1^

Reference	*n*	Participants^2^	Design	Intervention	Duration	Method of randomization	Results
Knopp et al. (47)	55	10–21 y; overweight	Parallel	2.7 g/d encapsulated aspartame vs. placebo during 1000 kcal/d diet	13 wk	Not specified	No significant differences in weight loss between 2.7 g/d encapsulated aspartame vs. placebo
Ebbeling et al. (48)	103	13–18 y; consuming ≥12 oz (355 mL) SSB/d	Parallel	Home delivery of noncaloric drinks (bottled water and LCSB) vs. usual beverage consumption	25 wk	Stratified, blocked randomization	No significant difference in BMI change between intervention group vs. control group; post hoc analysis showed greater BMI reduction with intervention in the tertile with highest baseline BMI (≥25.6 kg/m^2^)
Williams et al. (23)	32	11–15 y; obese girls	Parallel	1500-kcal/d diet with SSB permitted (free snack) vs. not permitted (restricted snack)	12 wk	Not specified	No significant difference in BMI in the 2 groups; free snack group consumed 3 ± 1.2 SSB/wk, and restricted snack group consumed 0.4 ± 0.9 LCSB/wk
Rodearmel et al. (49)	184	7–14 y; overweight	Parallel	Add 2000 steps/d + reduce 100 kcal/d (in part by using Splenda instead of sugar vs. usual lifestyle)	24 wk	Not specified	No significant difference in change in BMI Z-score between groups; more children in intervention group maintained or reduced BMI Z-score vs. control group

1 LCS, low-calorie sweetener; LCSB, low-calorie sweetener beverage; RCT, randomized controlled trial; SSB, sugar-sweetened beverage.

2 Ages are at study entry.

### LCS and weight gain prevention.

Hill et al. (50) have suggested that small behavior changes resulting in a deficit of 100 kcal/d may be sufficient to arrest weight gain in adults. It is important to note that children should experience weight gain with normal growth. As such, it is often inappropriate to promote weight loss in children aged <14 y. A preferred approach for growing children may be to focus on slowing down weight gain or maintaining weight as height “catches up.” Therefore, a small-changes approach, in which children increase energy expenditure by 100 kcal/d and decrease energy intake by 100 kcal/d, could create an energy gap (the difference between what is needed and what is spent). In addition, using small changes may help children to form sustainable healthy eating and active living habits that allow them to grow into their weight. Another advantage of creating a daily energy deficit of ≤200 kcal/d is that small perturbations in energy deficit do not result in the decreased metabolic rate typically associated with weight loss. Furthermore, eating 100 kcal fewer per day does not seem to increase feelings of hunger (51). Finally, incorporating small dietary and physical activity behavior changes seems to be easy and attainable for a variety of individuals (52).

Putting this small-change approach to work in children, the America On the Move Foundation, an evidence-based nonprofit organization, developed a program designed to help families increase daily activity and make healthier eating choices. A 2006 study by Rodearmel et al. (53) showed that the family program increased daily step activity (*P <* 0.0001) and positively affected the BMI-for-age (*P <* 0.05) of target children. The families recruited for this study had at least one child aged 8–12 y that had a BMI-for-age at or above the 85th percentile (target child).

Historic population data have positively correlated sugar consumption and obesity rates (54). In fact, SSB consumption has been shown to be independently related to increases in BMI over time in children (38). On average, it is estimated that children aged 2–19 y consume 10–15% of their daily energy as SSB or 100% fruit juice (55, 56). Substitution of just one sugar-sweetened beverage with a no- or LCSB per day provides one small-change approach that the America On the Move Family Program incorporates to decrease overall energy intake; as noted earlier, creating an energy deficit of 100 kcal/d may not lead to weight loss but instead prevention of excessive weight gain in children.

In 2007, Rodearmel et al. (49) assessed the America On the Move family program again but specifically asked families to increase daily steps by 2000 steps/d and to replace 100 kcal/d of dietary sugar with a nonenergy-containing sweetener (Splenda, McNeil Nutritionals). Families with at least one child aged 7–14 y who was ≥85% BMI-for-age were recruited to participate in the study (49). Over a 6-mo period, a significantly higher number of children who were randomized to the America On the Move family program maintained or reduced their BMI-for-age (*P <* 0.05).

A small-changes approach that incorporates easy tips, such as reducing energy intake by using a no-calorie sweetener or LCS and increasing daily activity by ~2000 steps (~100 kcal) may be a healthy, sustainable way to prevent and reverse childhood overweight and obesity.

### Unanswered questions regarding LCS use in children.

The best evidence about the effects of LCS on body weight comes from RCT. To date, results from small studies in adolescents have been consistent with studies in adults, showing that LCS are neutral to modestly effective in achieving short-term BMI reduction in overweight adolescents.^10^ The long-term effects of LCS in adolescents, however, are unknown. Although the body of evidence regarding LCS effects in adolescents is growing, very little information is available in younger populations. One of the key questions for pediatricians, parents, and other stakeholders is whether LCS have different effects at different developmental stages and thus whether recommendations about their use should vary across the lifespan. For example, young children seem to have a better ability to regulate energy intake compared with older children and adults (42). Thus, reduction of energy in certain foods and drinks using LCS in preschoolers may simply result in a compensatory increase in energy intake from other foods and drinks, leading to net energy balance. In addition, there is concern that the dissociation of the sense of sweetness and the energetic value of foods and drinks caused by LCS may result in overeating and weight gain, an effect that may be particularly relevant in young children. Although this theory is largely supported by animal data (56), one small study in children aged 3–5 y supports this hypothesis (45). Overall, there are insufficient data at this time to support the use of LCS in infants and young children and theoretical concerns exist about the potential of early exposure to LCS to affect a lifetime of eating habits. The National Children’s Study (57) holds the potential to elucidate patterns of LCS use and subsequent growth parameters from birth to age 21 y, potentially leading to well-designed interventional studies of LCS in children of varying ages.

Finally, new data are coming to light regarding the active role that LCS may play in the gastrointestinal tract. In vitro and animal studies have convincingly shown that sweet taste receptors, identical to those found in lingual taste buds, are located on enteroendocrine cells of the gastrointestinal tract (58–60). These receptors bind to LCS in addition to energy-containing sugars, resulting in secretion of the incretin hormone glucagon-like-peptide-1 (GLP-1) (60). The known effects of GLP-1 include delayed gastric emptying, increased insulin secretion, and suppressed appetite. In addition, sucralose has been shown in animal studies to upregulate intestinal glucose transporters, thus increasing the rate at which glucose is absorbed from the gastrointestinal tract into the bloodstream (61). Although few data are available in humans, one study showed increased GLP-1 secretion in participants aged 12–25 y following the ingestion of diet soda (62) and a similar study is ongoing in children aged 6–12 y (63).

## Summary and Conclusions

The prevalence of overweight and obesity among children in the United States remains high and is linked to a number of comorbidities. Authoritative medical and health organizations agree that lifestyle and dietary interventions should be considered in prevention and treatment. Research is needed to develop and implement interventions that are effective and sustainable. Observational studies tend to show positive associations between LCSB intake and BMI, suggesting that LCS alone may not be an effective strategy in preventing excess weight gain in children. However, limited data suggest that LCS used as one aspect of a multi-faceted program may be beneficial in preventing and reversing overweight and obesity. Additional, well-controlled research is needed to test this finding. The effectiveness for long-term weight maintenance in children has not been evaluated and very little related data exist for adolescents, although some compelling research is underway.

## Footnotes

8 Abbreviations used: AAP, American Academy of Pediatrics; GLP-1, glucagon-like-peptide-1; LCS, low-calorie sweeteners; LCSB, low-calorie sweetened beverage; RCT, randomized controlled trial; SSB, sugar-sweetened beverage.

9 www.clinicaltrials.gov; NCT00381160.

10 www.clinicaltrials.gov; NCT00250497.
